# Spatial Variability of Grapevine Bud Burst Percentage and Its Association with Soil Properties at Field Scale

**DOI:** 10.1371/journal.pone.0165738

**Published:** 2016-10-31

**Authors:** Tao Li, Xinmei Hao, Shaozhong Kang

**Affiliations:** 1 Center for Agricultural Water Research in China, China Agricultural University, Beijing, P. R. China; 2 State Key Laboratory Base of Eco-hydraulic Engineering in Arid Area (Xi'an University of Technology), Xi’an, Shannxi, P. R. China; UC Davis MIND Institute, UNITED STATES

## Abstract

There is a growing interest in precision viticulture with the development of global positioning system and geographical information system technologies. Limited information is available on spatial variation of bud behavior and its possible association with soil properties. The objective of this study was to investigate spatial variability of bud burst percentage and its association with soil properties based on 2-year experiments at a vineyard of arid northwest China. Geostatistical approach was used to describe the spatial variation in bud burst percentage within the vineyard. Partial least square regressions (PLSRs) of bud burst percentage with soil properties were used to evaluate the contribution of soil properties to overall spatial variability in bud burst percentage for the high, medium and low bud burst percentage groups. Within the vineyard, the coefficient of variation (CV) of bud burst percentage was 20% and 15% for 2012 and 2013 respectively. Bud burst percentage within the vineyard showed moderate spatial variability, and the overall spatial pattern of bud burst percentage was similar between the two years. Soil properties alone explained 31% and 37% of the total spatial variation respectively for the low group of 2012 and 2013, and 16% and 24% for the high group of 2012 and 2013 respectively. For the low group, the fraction of variations explained by soil properties was found similar between the two years, while there was substantial difference for the high group. The findings are expected to lay a good foundation for developing remedy measures in the areas with low bud burst percentage, thus in turn improving the overall grape yield and quality.

## Introduction

Grapevine (*Vitis spp*.) is one of the most cultivated crops in the temperate regions of 30°and 50°latitude [1] and vineyard is inherently spatially heterogeneous due to highly variable soil properties within a field. With the development of global positioning system (GPS), geographical information system (GIS), and crop yield monitor, there is an increasing interest for growers to adopt precision agriculture management practices, or precision viticulture when applied on grapevine[2–3]. Knowledge on spatio-temporal pattern of within vineyard variation in grape yield, quality or associated components is essential for practicing any targeted precision viticulture measures. Therefore, study on spatial variability of grape yield, quality or other associated traits and its possible association with soil properties would help identify less productive area, which in turn facilitate developing site-specific management practices to optimize yield and/or quality in those areas and ultimately achieve the best return over the whole field.

Spatial variability in grape yield and berry composition at a field scale and their potential association with soil and topographic properties have been widely investigated. Substantial spatial variability in grape yield was found within three Australian vineyards of 3.6, 4.5 and 7.3 ha with the highest yield about 8–10 fold of the lowest yield at any given site [3]. They also found that spatial pattern of grape yield was fairly stable over the three-year period. Coefficient of variation of grape fruit compositions at harvest, including in juice pH, anthocyanins, phenolics, titratable acidity and Baumé, ranged from 2.8 to 21.6% in a 7.3 ha vineyard [4]. Tisseyre et al. [5] studied the temporal stability of spatial variability in several vine parameters including pruning weight, yield, canopy size, sugar, total titrable acidity, and pH based on consecutive 7-year field experiments. On the other hand, Tardaguila et al. [6] constructed a soil index from four soil variables (thickness of the top soil layer containing 80% of the visible roots, soil organic matter content, clay content, and cation exchange capacity) and found that vine yield parameters such as yield per vine, berry weight, and cluster weight were significantly correlated with the areas delineated based on the constructed soil index. Morlat and Bodin [7] compared the differences in grape yield and berry quality on the different terroir units defined based on soil depth, clay content and weathering degree of the parent rock. Bramley et al. [8] attempted to build the associations between vineyard soil properties with grape or wine attributes and found that high yield zone tended to have relatively higher level of soil extractable Fe. Previous studies have reported that grape yield was determined mostly by the bunch number per vine and the berry number per bunch, which were shown to account for 60% and 30% of seasonal yield variation respectively [9–11]. Therefore, bud behavior may be closely related to grape yield. Bud burst percentage has been often employed as an important index for studying different treatment effects on the status of vine vigour in the early season. For example, Elgendy et al. [12] investigated the effects of foliar spraying of gibberellic acid and/or forchlorfenuron on bud burst percentages (ratio of burst buds over the total number of buds per vine) and fruitful buds of Thompson Seedless grape based on three-year experiments. Guilpart et al. [9] examined the association of bud fertility (number of bunches per shoot) with soil water status during different time period of the current and the previous season for different irrigation and nitrogen treatments.

However, spatial pattern of bud bust percentage is not expected to resemble the spatial pattern of yield or fruit composition, considering that bud bursting occurs at early growing season, and final yield or fruit quality are affected by many other factors at later growing period, such as climate and viticulture practices. To our knowledge, there were few reports on spatial variation in bud burst percentage until present. Knowledge of spatial variation in bud burst percentage and at what extent soil properties contributing to the variation is relevant for developing precision viticulture practices during the bud bursting period that intend to achieve optimal growing conditions during the time of period. The objective of this study was to investigate spatial variability of bud burst percentage and its association with some selected soil properties using partial least square regression method based on 2-year experiments at a vineyard located in arid northwest China.

## Materials and Methods

### Field description and data collection

**Fig 1:**
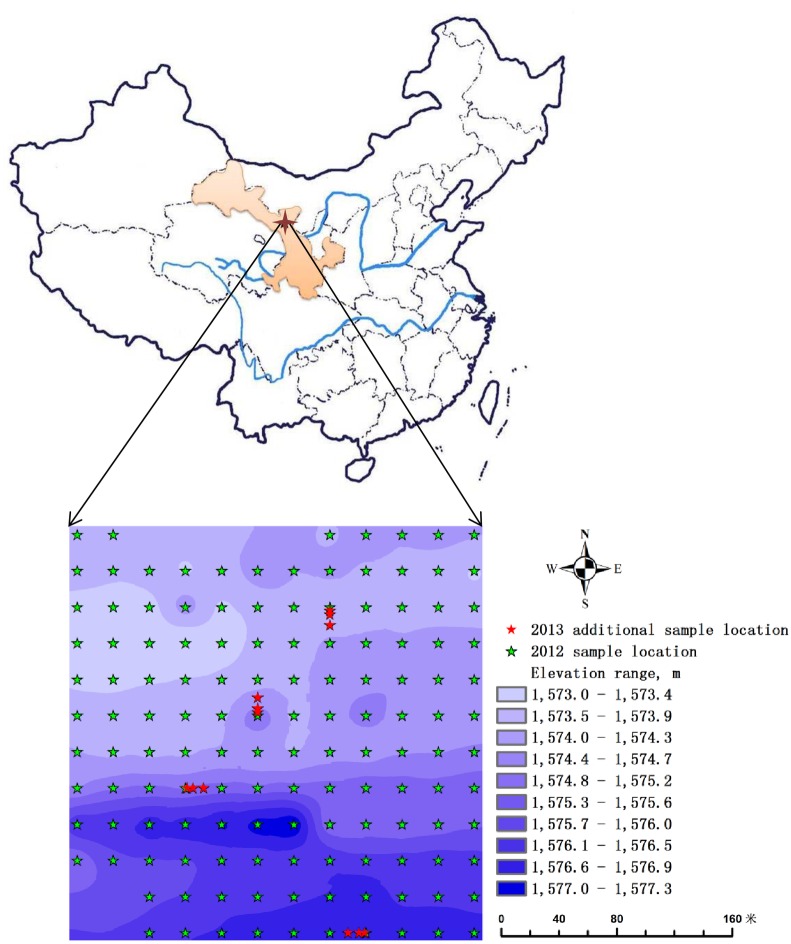
The location of the experimental site in the country, along with layout of the sampling location and elevation map of the field.

**Fig 2:**
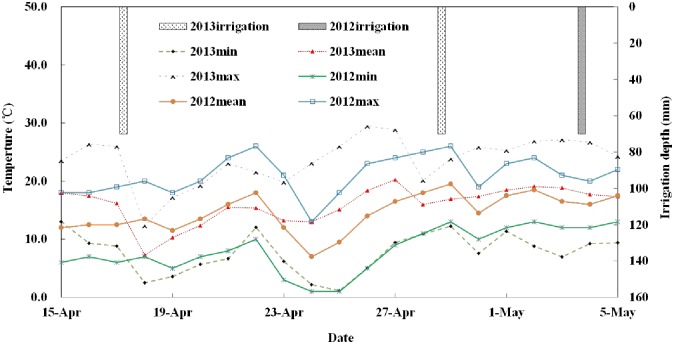
Daily maximum, mean and minimum temperatures and irrigation water amount during the bud burst period in 2012 and 2013.

The experiment was conducted in Huangtai wine vineyard with an area of 7.6 ha at the Shiyanghe Experimental Station for Improving Water Use Efficiency in Agriculture, Ministry of Agriculture (MOA), located at Wuwei City of Gansu Province (N 37°52´20″, E 102°50´50″, and altitude 1581 m) from April 15 to May 5, in 2012 and 2013. Fig 1 shows the location of the experimental site in the country. This region is located in a typical continental temperate arid climate zone with hot summer, cold winter, dry and windy weather. Mean annual precipitation of the region is about 164 mm, and mean annual pan evaporation is about 2000 mm. The maximum, mean, and minimum daily temperatures were measured using a standard automatic weather station at the experimental station during the bud burst period. The temperature was similar during the bud burst period of the two years (Fig 2).

Measurements of number of burst buds were taken at regularly-spaced points with 25-m apart at both east-west and north-south directions in a 275×275 m area. There were 9 points at northwest and southwest edges were omitted from the study since corn instead of grapevine was mistakenly planted in those areas in both years. As a result, there were a total of 135 points in 2012 and 147 points for 2013 after additional 12 points were added as Fig 1 has shown.

The grape variety used in the study was *Vitis vinifera* L. cv Merlot Noir, which was established in 1999 at a spacing of 270 cm between rows oriented east–west and 100 cm between vines. Vines were trained to a vertical plane by every other about 9 m pillars with three wires supported by a 1.5 m high trellis. Each vine was spur pruned to 2 or 3 nodes per spur several days earlier before being buried, usually at the end of leaf fall period. About one month after bud burst, every new shoot was removed top and there were one or two flower buds and about 10 leaves per shoot remaining after the removal. Usually the vineyard is furrow-irrigated 4 or 6 times during the growth period of each year, roughly in early May, late May, early July and late August, and for each irrigation event, about 70 mm water is applied. Before each irrigation event, fertilizers usually including urea, zinc compound fertilizer, boric acid compound fertilizer and phosphate diamine were applied into holes between grapevines. During the bud burst period, irrigation was only applied once on May 4 of 2012, while twice on April 17 and April 29 of 2013 (Fig 2). The detailed information about the studied site and grapevine were described in literature [13]. All field management practices were the same over the entire vineyard.

At the end of bud burst period, number of burst buds and total number of buds were counted on randomly selected 3 healthy grape vines around each sampling point. For burst buds or total number of buds, only primary ones were counted. Bud burst percentage (BB) was calculated as follows:

$$\text{BB}=\frac{number\;of\;burst\;buds\;per\;vine}{total\;number\;of\;buds\;per\;vine}\times 100\text{\%}$$ (1)

Soil samples were taken at those 135 points in May of 2012, and two soil subsamples collected at the surrounding area of the point were mixed for 0–20 cm and 20–40 cm depth respectively. For each soil sample, total nitrogen (TN), total phosphorus (TP), total potassium (TK), organic carbon (OC), Ca, Mg, clay, silt, sand, and bulk density (BD) were measured at Soil Chemistry Laboratory of Northwest Agriculture and Forestry University of China. Specifically, TN was determined using the Kjeldahl method [14], TP was measured by molybdenum-antimony anti-spectrophotometric method [15], TK was measured by alkali fusion and flame photometer method [16], and Mg^2+^ and Ca^2+^ were measured using atomic absorption spectrophotometry [17–18]. Soil organic carbon content was measured using the potassium dichromate method [19–20] and BD was determined gravimetrically using oven drying method. Soil texture (clay, silt, and sand content) was measured using a MaterSizer2000 laser particle size analyzer (Malvern Instruments Ltd., U.K).

### Data analysis

Spatial structure of bud burst percentage was evaluated by fitting sample variogram with three commonly used empirical models: spherical, exponential and Gaussian model. Details on variogram calculation and model description could be found in a number of literatures [21–24]. The fitting accuracy of model was evaluated by the determination coefficient (*R*^2^) and the residual sums of squares (RSS). Semivariogram calculations and corresponding model fittings were conducted using GS+9.0 software [25].

According to Legendre [26] and Lichstein et al. [27], the total variation of a spatial variable could be decomposed into spatially structured variation shared by environmental variables, spatial structured variation not shared by environmental variables, non-spatially structured variation explained by environmental variables, and unexplained variations. The spatially structured variation obtained by the partition method was referred to as broad-scale spatial dependence, contrary to fine-scale spatial dependence or autocorrelation assumed in geostatistical analysis [27]. Knowledge of each fraction could help better understand the extent of environmental factors contributing to the variable’s total variation. In this study, environmental variables were referred to the measured soil properties only. Topographic attributes were not included due to the narrow range of elevation over the field and insignificant correlation between bud burst percentage with topographic attributes. To partition the variation in practice, regression models were built between the spatial variable, bud burst percentage in this study, with soil properties alone, polynomial terms of spatial coordinates, and combination of the two sets of variables, respectively. The regression model based on the soil property variable alone was referred as the environment model, the regression model based on polynomial terms of spatial coordinates was referred as the trend surface model, and the regression model based on the combination of soil variables and spatial coordinate terms was referred as the full model. Therefore, each of the four fractions of the total variation was obtained based on the *R*^2^ of the three regression models mentioned above according to the method employed by Lichstein et al. [27]. In the study, partial least square regression (PLSR) analysis was performed to build the regression models mentioned to avoid the potential multicollinearity problem in the regression analysis.

To evaluate the possible difference of those fractions for different levels of bud burst percentage, bud burst percentage data were classified into three groups according to their magnitude, i.e. low group when bud burst percentage was less than its first quartile, high when it was larger than the third quartile, and medium for those between the first and third quartile. Then similar regression analyses were conducted separately for the low, medium and low groups. Pearson correlation coefficients were calculated between the first and second extracted factors from PLSRs of bud burst percentage with the soil properties for the three groups for both years of 2012 and 2013. PLSR analyses and Pearson correlation coefficient calculation were conducted using SAS 9.1.3 (SAS Institute, 2004) [28].

## Results and Discussion

### Description statistics of bud burst percentage

Table 1 shows the number of samples, maximum, mean, minimum, standard deviation, and CV of bud burst percentage in 2012 and 2013, as well as descriptive statistics of the soil properties. Bud burst percentage showed great variation in the field, ranging from 18.8 to 75.0% in 2012, and from 10.2 to 81.0% in 2013, respectively. The mean bud burst percentage was 58.1% in 2013, higher than in 2012 with mean value of 50.0%, while CV was lower in 2013 than in 2012, probably due to wetter and more uniform distribution of soil water content in 2013 given that there were two irrigation events in 2013, compared to only one in 2012. These numbers were comparable to previous results reported by Kubota and Miyamuki [29] who found that the averaged bud burst percentage for Kyodo, Delaware and Neo Muscat varieties were 55.6%, 66.7% and 66.2%, respectively. Elgendy et al. [12] found that bud burst percentage was roughly within 60–70% under different foliar spraying treatments. Soil properties in the field showed weak to moderate variability in the field, with the highest CV around 34.6% for TP and sand content, and the lowest CV of 4 and 7% for bulk density and TK, respectively.

**Table 1:** Description statistics of grapevine bud burst percentage in 2012 and 2013, as well as of the statistics of soil properties in the vineyard.

	N	Minimum	Maximum	Mean	Std	CV(%)
BB_2012(%)	135	18.78	75.00	50.02	10.02	20.04
BB_2013(%)	147	10.20	80.95	58.14	9.62	16.54
TN(%)	135	0.02	0.08	0.05	0.01	21.28
TP(%)	135	0.03	0.20	0.10	0.04	34.65
TK(%)	135	1.35	2.08	1.74	0.13	7.49
OC(%)	135	0.40	1.38	0.81	0.17	20.98
Ca^2+^(mg/kg)	135	2387.54	4615.75	3503.00	455.62	13.01
Mg^2+^(mg/kg)	135	200.07	515.84	355.44	64.11	18.04
Clay(%)	135	9.63	22.16	15.99	2.36	14.76
Silt(%)	135	52.98	75.51	67.54	3.91	5.80
Sand(%)	135	6.25	36.94	16.46	5.71	34.67
BD(g/cm^3^)	135	1.39	1.72	1.56	0.07	4.36

BB_2012 and BB_2013: bud burst percentage in 2012 and 2013, respectively; Std: standard deviation; CV: coefficient of variation; TN: total nitrogen; TP: total phosphorus; TK: total potassium; OC: organic carbon; BD: bulk density.

### Geostatistical analysis of spatial variation of bud burst percentage

**Fig 3:**
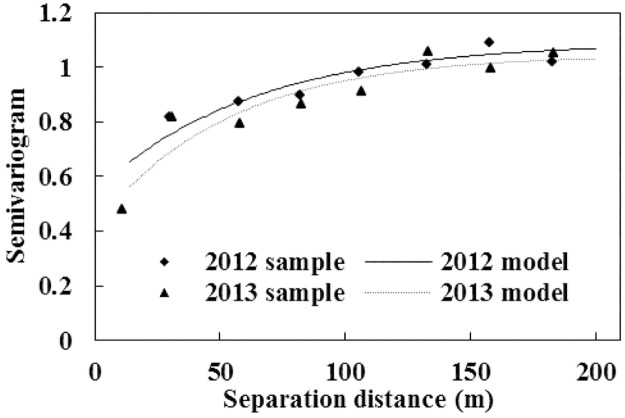
Calculated variograms and fitted models for the standardized bud burst percentage data in 2012 and 2013, respectively.

**Fig 4:**
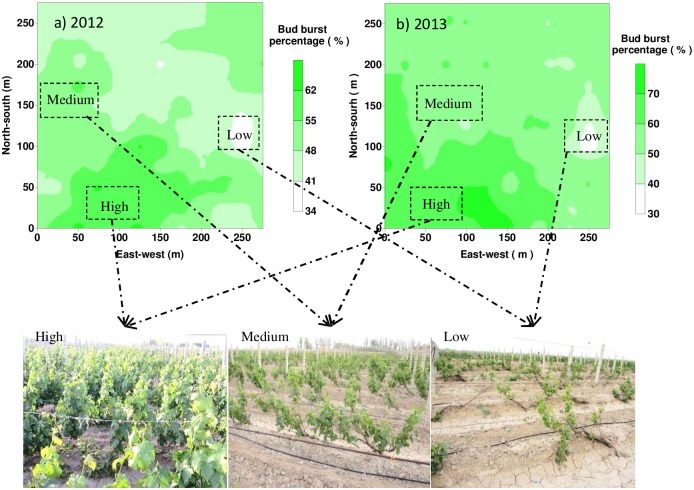
Spatial distribution of bud burst percentage obtained through ordinary kriging: (a) in 2012; (b) in 2013; Pictures for the low, medium and high groups are also shown and all pictures were taken on May 18, 2013.

Relative to the other variogram models, exponential model fitted the computed sample variogram better with the largest *R*^2^ and smallest RSS. Fig 3 displays the sample variogram values along with the fitted model for both 2012 and 2013 data, while fitted model parameters are shown in Table 2. The difference in fitted sill value between 2012 and 2013 could be attributed to different sampling plan used in 2013 when additional sampling points with closer distance were added. Apparently, variograms of bud burst percentage were very similar for the two years, with the values in 2012 slightly larger than those in 2013. Spatial distribution of low, medium, and high bud burst percentage zones, created based on ordinary kriging, displayed great similarities over the two years as shown in Fig 4. In both years, bud burst percentage seems to be higher at the southwest corner while lower at the middle of east part of the study site. The similar spatial distribution map and variogram model indicated spatial variation pattern of grapevine growth was stable over the two years, which was consistent with the results of Bramley and Hamilton [3, 30]. The stable spatial variation pattern lays a very good foundation for applying site-specific or spatially variable-rate management practices in the field. According to Bramley and Hamilton [3], the consistency of within-field spatial variation pattern from year to year should be essential for adopting any precision agriculture management measures.

**Table 2:** Parameters of the fitted variogram models for the standardized bud burst percentage data in 2012 and 2013, respectively.

Year	Model	C_0_	C_0_+C	Range/m	DSD	R^2^	RSS
2012	Exponential	0.55	1.09	188.7	0.50	0.86	0.01
2013	Exponential	0.42	1.05	159.0	0.40	0.88	0.03

C_0_: nugget; C_0_+C: sill; DSD: degree of spatial dependence, C_0_/ (C_0_+C); R^2^: the determination coefficient; RSS: the residual sums of squares.

### Correlation between bud burst percentage and soil properties

Pearson’s correlation coefficients between soil properties and bud burst percentage for high, middle, and low zones, as well as for the entire field are shown in Table 3. There was significant positive correlation between the bud burst percentage in 2012 and 2013 (*R* = 0.467, p < 0.01), suggesting that to some extent, bud burst percentage was influenced by temporally invariant factors in the field, most likely soil properties. Overall, bud burst percentage in 2012 was found significantly and negatively correlated with soil Ca, Mg, and clay contents, and positively with sand content, while bud burst percentage in 2013 was not significantly correlated with any soil variables but soil clay content. Similar results showed yield on clayey and gravelly soil was 32 and 62% lower than that on sandy, respectively, from 1996 to 2000 in three Saint-Emilion vineyards in France[31]. For the low group of both years, but burst percentage was found negatively correlated with TP. Previous studies have shown that P fertilization generally increased grapevine vigour, such as berry weight, pruning weight and yield [32]. Therefore, low bud burst percentage in the low zone was not likely the causal effect of high TP. Some other unfavorable conditions for bud bursting that were correlated with TP might contribute to the low bud burst percentage. Further studies are needed to pinpoint the limiting factors affecting bud burst in the vineyard. For the high group, soil properties were found not significantly correlated with bud burst percentage in both years except soil bulk density in 2013, suggesting that soil properties might not be the most important factors determining the spatial variability of bud burst percentage within the high group.

**Table 3:** Pearson’s correlation coefficients between bud burst percentage of 2012 and 2013 with soil properties for low, medium and high groups respectively (numbers significant at 0.05 level only).

	BB_2012			BB_2013			BB_2012	BB_2013
	Low n = 33	Medium n = 69	High n = 33	Low n = 32	Medium n = 71	High n = 32	n = 135	
BB_2012								0.467**
BB_2013							0.467**	-
TN								
TP	-0.397*			-0.467**				
TK				-0.406*				
OC								
Ca		-0.296*					-0.308**	
Mg							-0.282**	
Clay		-0.327**					-0.179*	-0.182*
Silt								
Sand							0.183*	
BD						-0.616**		

BB_2012 and BB_2013: bud burst percentage in 2012 and 2013, respectively; TN: total nitrogen; TP: total phosphorus; TK: total potassium; OC: organic carbon content; BD: bulk density;

*, ** significant at the 0.05 and 0.01 probability level, respectively;

- non-applicable.

### Partitioning spatial variation in bud burst percentage

**Fig 5:**
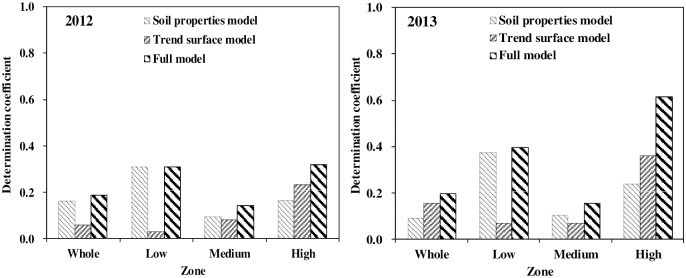
Determination coefficient (R^2^) from partial least square regressions of bud burst percentage with soil properties, trend surface model, and combination of the two sets of variables for the low, medium and high groups as well as for the entire field of 2012 and 2013 seasons.

Coefficients of determination (*R*^2^) from PLSRs with different sets of variables are shown in Fig 5 for the low, medium, and high groups, and the entire field of the two years. From PLSRs over the entire vineyard, the full model explained less than 20% of the overall bud burst percentage variation in both years, indicating bud burst percentage variation over the vineyard was not well accounted for by the variables included in the model. For the low group, the explained variations by the soil properties, trend surface, and the full model were similar between the two years, while for the high group, the explained variations of the three models were apparently higher in 2013 than those in 2012. We speculate that low bud burst percentage was determined to some extent by negative impacts of some intrinsic properties, such as soil properties, and more importantly, the accumulated impacts of those factors over the years on the growth of vines, as Fig 4 has shown that vines themselves appeared to be weaker in the low group. Therefore, the explained variations by soil properties were quite consistent for the low group between the two years. On the other hand, factors contributing to the high bud burst percentage might be different according to different environmental conditions, which led to large differences in *R*^2^ for all three models between the two years. It is noticeable that *R*^2^ from all three sets of variables was apparently lower for the medium groups probably due to difficulties in building a strong relationship between but burst percentage with the complex factors which might have opposite effects in the medium group.

**Fig 6:**
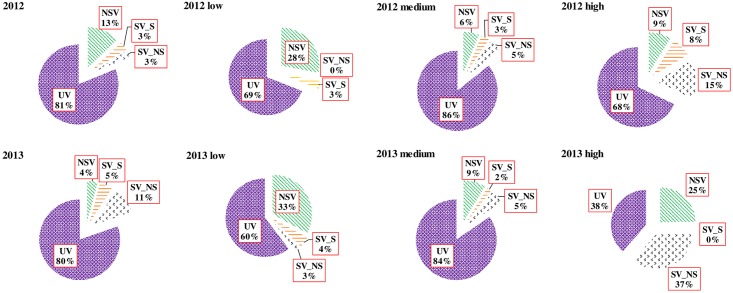
Partition of the overall spatial variation into nonspatial soil variation (NSV), spatially structured variation shared by soil variables (SV_S), spatial variation not shared by soil variables (SV_NS) and unexplained variation (UV). The calculations were based on the partial least square regressions of bud burst percentage with soil, trend surface, and combination of the two sets of variables.

Fractions of the total variation, i.e. nonspatial soil variation(NSV), spatially structured soil variation(SV_S), spatial variation not shared by soil properties(SV_NS), and unexplained variation(UV), were calculated based on PLSRs with different sets of variables (Fig 6). The fractions of variation were shown quite similar for the low and medium groups between the two years while there were substantial differences for the high group. For the low and medium groups, the majority of variation was not explained by either the soil variables or spatial coordinate terms with UV up to 69 and 86% for the low group in 2012 and medium groups in 2013, respectively. Previous study has suggested that the effect of climate is more important in determining the grape yield, relative to soil properties [31]. Therefore, spatial variability in microclimate or soil variables other than those included in the model might to a greater extent determine the spatial variability in bud burst percentage.

Meanwhile, broad-scale spatial variation in bud burst percentage for the low and medium groups was less than 8% in both years while NSV accounted for most of the explained variation for the low group, 28 and 33% for 2012 and 2013 respectively. Relative to the other two groups, fractions of the total variation showed a different pattern for the high group, particularly in 2013. In 2013, the majority of the variation actually could be explained by the combined soil variables and spatial coordinates with UV of 38% for the high group while NSV (25%) and SV_NS (37%) accounted for the explained variation. It seems that large portion of the broad-scale spatial variation in bud burst percentage was attributable to soil properties for the low group while for the high group, large portion of broad-scale spatial variation was most likely not from the included soil properties in both years. The results are expected as unfavorable soil conditions, such as water or nutrients deficiency, often affect crop growth negatively, thus are the important factors resulting in weaker growth status of crops, lower bud burst percentage in this case. For the high group, growing conditions are generally more favorable and soil properties become less pronounced in determining spatial variation in vines’ growth status.

To further examine the association between soil properties and bud burst percentage, Table 4 shows Pearson correlation coefficients between the extracted factors from PLSRs of bud burst percentage with soil properties alone (environmental model) for the low and high groups. For the low group, the first and second factor of 2012 were found significantly positively correlated with the respective first and second factors of 2013 with the correlation coefficients of 0.65 and 0.5, respectively, suggesting the underlying factor from soil properties resulting in low bud burst percentage was somewhat consistent over the two-year period. For the high group, the first and second factor of 2012 were found not significantly correlated with the first and second factor of 2013 accordingly. However, the first and second factor of 2012 were significantly correlated with the second and first factor of 2013 with correlation coefficients of 0.8 and 0.5, respectively, suggesting that the combined effects of soil properties contributing to the high bud burst percentage seems to be consistent, although the relative importance of the factors might be different over the two years. Therefore, application of PLSR method, relative to building the model with individual soil properties, could be a useful tool to identify underlying factors that tend to be more informative and consistent.

**Table 4:** Pearson’s correlation coefficients between the extracted factors from partial least square regressions (PLSRs) of bud burst percentage with soil properties for the low and high groups of 2012 and 2013 respectively. Two factors were selected for each PLSR.

	Y12_L1†	Y12_L2	Y13_L1	Y13_L2	Y12_H1	Y12_H2	Y13_H1	Y13_H2
Y12_L1	1							
Y12_L2	0	1						
Y13_L1	0.65***	-0.72***	1					
Y13_L2	0.45**	0.51**	0	1				
Y12_H1	-0.17	0.27	-0.29	-0.16	1			
Y12_H2	-0.01	0.36*	-0.33	0.33	0	1		
Y13_H1	0.03	0.64***	-0.45**	0.41*	-0.13	0.50**	1	
Y13_H2	-0.24	0.27	-0.33	-0.20	0.81***	-0.25	0	1

^†^Y12(13)_L(H)1(2) indicates the first (second) factor for the low (high) group of year 2012 (2013);

*, **, and *** indicate significance level at 0.05, 0.01, and 0.001, respectively.

## Conclusions

Bud burst percentage showed considerable spatial variability within a 7.5 ha vineyard with its values ranging between 8.8–75.0% in 2012, and between 10.2–81.0% in 2013, respectively. The calculated sample variograms of bud burst percentage and fitted vairogram models were similar between the two years. Coefficients of determination (*R*^2^) from partial least square regression of bud burst percentage with soil properties were 0.31 and 0.37 for the low bud burst percentage group of 2012 and 2013, and 0.16 and 0.24 for the high percentage group of 2012 and 2013 respectively. Addition of spatial coordinate terms did not improve the *R*^2^ for the low group of both years, but improved *R*^2^ to 0.32 and 0.61 for the high group of 2012 and 2013 respectively. The extracted factors from the PLSRs with soil properties in 2012 were found significantly correlated with those in 2013 for both groups. Over the two years, the variation explained by soil properties on bud burst percentage tended to be consistent for the low group while the fraction of variations tended to be more variable for the high group.

## Supporting Information

S1 Table Bud burst percentage, elevation, soil properties and relative coordinates at sampling locations, along with weather information at the vineyard. (XLSX)
